# Comparative Functional Genomics Analysis of NNK Tobacco-Carcinogen Induced Lung Adenocarcinoma Development in *Gprc5a*-Knockout Mice

**DOI:** 10.1371/journal.pone.0011847

**Published:** 2010-07-29

**Authors:** Junya Fujimoto, Humam Kadara, Taoyan Men, Carolyn van Pelt, Dafna Lotan, Reuben Lotan

**Affiliations:** 1 Department of Thoracic/Head and Neck Medical Oncology, University of Texas M.D. Anderson Cancer Center, Houston, Texas, United States of America; 2 Department of Veterinary Medicine and Surgery, University of Texas M.D. Anderson Cancer Center, Houston, Texas, United States of America; Emory University, United States of America

## Abstract

**Background:**

Improved understanding of lung cancer development and progression, including insights from studies of animal models, are needed to combat this fatal disease. Previously, we found that mice with a knockout (KO) of G-protein coupled receptor 5A (*Gprc5a*) develop lung tumors after a long latent period (12 to 24 months).

**Methodology/Principal Findings:**

To determine whether a tobacco carcinogen will enhance tumorigenesis in this model, we administered 4-(methylnitrosamino)-1-(3-pyridyl)-1-butanone (NNK) i.p. to 2-months old *Gprc5a*-KO mice and sacrificed groups (n = 5) of mice at 6, 9, 12, and 18 months later. Compared to control *Gprc5a*-KO mice, NNK-treated mice developed lung tumors at least 6 months earlier, exhibited 2- to 4-fold increased tumor incidence and multiplicity, and showed a dramatic increase in lesion size. A gene expression signature, NNK-ADC, of differentially expressed genes derived by transcriptome analysis of epithelial cell lines from normal lungs of *Gprc5a*-KO mice and from NNK-induced adenocarcinoma was highly similar to differential expression patterns observed between normal and tumorigenic human lung cells. The NNK-ADC expression signature also separated both mouse and human adenocarcinomas from adjacent normal lung tissues based on publicly available microarray datasets. A key feature of the signature, up-regulation of *Ube2c*, *Mcm2*, and *Fen1*, was validated in mouse normal lung and adenocarcinoma tissues and cells by immunohistochemistry and western blotting, respectively.

**Conclusions/Significance:**

Our findings demonstrate that lung tumorigenesis in the *Gprc5a*-KO mouse model is augmented by NNK and that gene expression changes induced by tobacco carcinogen(s) may be conserved between mouse and human lung epithelial cells. Further experimentation to prove the reliability of the *Gprc5a* knockout mouse model for the study of tobacco-induced lung carcinogenesis is warranted.

## Introduction

It has been estimated that there will be nearly 220,000 new cases and 160,000 deaths from lung cancer in the United States in the year 2009 [1]. Non small-cell lung cancer (NSCLC) represents the majority of lung cancers and includes two major histological subtypes; squamous cell carcinomas and adenocarcinomas [2]. The incidence of the latter type is increasing globally and in the United States [3], [4]. In addition, nearly 85% of newly diagnosed NSCLC patients are ever smokers clearly implicating tobacco and tobacco-related carcinogens in the etiology of NSCLC [5].

Lung carcinogenesis occurs through a multi-step process, which involves both, the activation or over-expression of oncogenes, and the inactivation or suppression of tumor-suppressor genes [2]. Mouse models for human lung cancer have proven to be valuable tools for understanding the basic tumor biology as well as for the development and validation of new approaches to cancer prevention and therapy [6]. While very few mouse strains exhibit spontaneous lung tumor development, an increasing number of models has been developed either by treatment with tobacco carcinogens [7], [8], [9], [10], [11], [12] or tobacco (or cigarette) smoke [13], [14], [15] as well as by introduction of mutant oncogenes or transgenes such as mutant *Kras* [16] or epidermal growth factor receptor (*Egfr*) [17], [18] or a combination of transgenes and carcinogens [19]. We have previously shown that mice with deletion of both alleles of the *Gprc5a* gene develop spontaneous lung adenomas and adenocarcinomas between the ages 12 and 24 months indicating that this gene is a mouse lung-specific tumor suppressor [20]. The *Gprc5a* knockout mouse was genetically engineered using the C57BL/6×129sv mixed background [20]. It is noteworthy that mice of each of these two parental strains were reported to be resistant to the carcinogenic effects of NNK [21], [22] compared to mice of the A/J strain, which have been shown to be predisposed to develop spontaneous lung adenomas and to be susceptible to NNK-induced lung tumorigenesis [11], [23]. Therefore, by analogy, we hypothesized that the knockout of *Gprc5a*, which predisposes mice to spontaneous lung tumorigenesis [20] will also sensitize them to a tobacco-specific carcinogen such as NNK. In addition, given the pivotal role of tobacco use in the etiology of human lung cancers [2], [4], we thought that if a tobacco carcinogen were to enhance lung tumorigenesis in the *Gprc5a* KO mice, this model would be more relevant to the human disease.

In this study, we show that exposure of *Gprc5a*-knockout mice to NNK tobacco-specific carcinogen increased the incidence and multiplicity of lung tumors, especially adenocarcinoma, compared to control mice. In addition, comparative functional genomics approaches have enabled us to demonstrate that the gene expression patterns signifying tobacco-carcinogen induced lung tumorigenesis may be conserved between mouse and human lung epithelial cells.

## Materials and Methods

### Chemicals and reagents

The tobacco carcinogen NNK with a purity of greater than 99% was purchased from Midwest Research Institute (The National Cancer Institute's Chemical Carcinogen Reference Standard Repository, Kansas City, MO). Mice were fed Purina chow #5001 (Harlan-Teklad, Madison, WI). Hematoxylin and Eosin staining reagents were purchased from DAKO (Carpinteria, CA) and Sigma-Aldrich (St. Louis, MO), respectively.

### NNK-induced tumorigenesis experimental design

We used (129sv× C57BL/6) F1 *Gprc5a* knockout (*Gprc5a* ^−/−^) mice, which were generated as described previously [20]. The mice were maintained according to a protocol approved by the M.D. Anderson Cancer Center Institutional Animal Care and Use Committee at the institution's specific pathogen-free animal facility, which is approved by the American Association for Accreditation of Laboratory Animal Care and is operated in accordance with current regulations and standards of the US Department of Agriculture and the Department of Health and Human Services.

**Figure 1:**
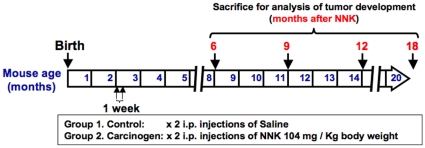
NNK-induced lung carcinogenesis in *Gprc5a*-knockout mice. Schematic illustration depicting the experimental plan where 2 months old *Gprc5a*-knockout mice were treated with 104 mg/kg body weight NNK or control saline intraperitonealy (i.p.) twice one week apart and followed up for lung lesion development at 6, 9, 12 and 18 months following treatment.

*Gprc5a* knockout mice (2 months old) were divided into groups of 5 or 6 mice and were injected twice (one week apart) intraperitonealy with NNK (104 mg/kg of body weight) dissolved in saline (0.9% NaCl) or with saline alone as control. Mice were selected randomly at 6, 9, 12 and 18 months after the second injection and sacrificed (Fig. 1A). The lungs were excised and inflated by injection of formalin and lung surface lesions were detected and enumerated by macroscopic observation. The lungs were then embedded in paraffin and histological sections were prepared and analyzed using standardized criteria [24] for characterization and diagnosis of lung lesions by two experienced pathologists (J.F. and C.V.P). The size of lung lesions was measured and quantified by the Image J program developed by the National Institutes of Health (rsbweb.nih.gov/ij/).

### *Gprc5a* ^−/−^ normal epithelial cells and MDA-F471 NNK-induced adenocarcinoma cells

Epithelial cells were derived from tracheas of 3-weeks old *Gprc5a* ^−/−^ mice [20] and MDA-F471 mouse lung adenocarcinoma cells were derived *de novo* from an adenocarcinoma that was found in the lung of a female *Gprc5a*-knockout mouse sacrificed 16 months after NNK i.p. injection based on a protocol approved by the M.D. Anderson Cancer Center Institutional Animal Care and Use Committee. Normal tracheas and lung adenocarcinoma dissected from the mice were excised, washed in antibiotic-containing phosphate-buffered saline (PBS), cut into small fragments (1–3 mm^3^) and suspended in a pre-warmed cell aggregate dissociation solution containing protease, collagenolytic and DNase activities (ACCUMAX™, Innovative Cell Technologies, San Diego, CA). The tissue fragments were then incubated for 1 hour at 37°C with occasional agitation and observation under the microscope to assess the efficacy of tissue disaggregation. Cell viability was assessed using the trypan blue dye exclusion method. Following complete disaggregation, cells were transferred to sterile 15 ml tubes and centrifuged at 300× g to pellet the cells and remove the ACCUMAX™ solution. The pellets were then resuspended in 1 ml of A_MNIO_MAX™ –C100 Basal Medium (GIBCO) containing A_MNIO_MAX™ –C100 Supplement (GIBCO). Cells were then seeded in 60 mm PRIMARIA™ tissue culture dishes (BD Biosciences, San Jose, CA) and maintained in culture using keratinocyte-serum-free medium (K-SFM) (GIBCO; Invitrogen, Grand Island, NY) supplemented with EGF (5 ng/ml) and Bovine pituitary extract (50 µg/ml) (GIBCO, Invitrogen) for the normal cells or DMEM:F12 medium supplemented with 10% fetal bovine serum for the MDA-F471 adenocarcinoma cells. The medium was replaced every 48 hours. All cells were grown at 37°C in a humidified atmosphere of 95% air and 5% CO_2_. The MDA-471 cells were tumorigenic as evidenced by the formation of tumors after injection of cultured cells (4×10^6^ cells in 10% Matrigel in PBS) subcutaneously into syngeneic mice. In contrast, the *Gprc5a* ^−/−^ normal lung epithelial cells isolated from normal tracheas were non-tumorigenic but formed viable colonies in soft agar.

### RNA extraction

Total RNA was purified from the MDA-F471 mouse adenocarcinoma cells using the RNeasy Mini kit according to the manufacturer's instructions (Qiagen, Valencia, CA). Total RNA was treated with DNase to eliminate genomic DNA. Purified total RNA was then quantified using the Nanodrop ND-1000® spectrophotometer (Thermo Fisher Scientific, Waltham, MA). RNA quality was assessed based on the 28S/18S ribosomal RNAs ratio using the Experion automated electrophoresis system (Bio-Rad Laboratories, Hercules, CA) according to the manufacturer's instructions.

### Microarray sample preparation, hybridization, scanning, and analysis

Double-stranded cDNA was synthesized with the Superscript Choice system (Invitrogen) using 5 µg of total RNA, isolated from the *Gprc5a* ^−/−^ MDA-F471 cells, for each strand and cleaned by phenol/chloroform extraction and ethanol precipitation. Biotin-labeled cRNAs were then synthesized by *in vitro* transcription (IVT) reaction using the ENZO BioArray High Yield RNA transcript labeling kit (Affymetrix, Santa Clara, CA). After cleanup of cRNA using RNeasy spin columns (Qiagen), fragmented cRNAs were hybridized to GeneChip® Mouse Genome 430 2.0 arrays (Affymetrix) according to the manufacturer's instructions. The arrays were scanned with a GeneChip® Scanner 3000 from Affymetrix and raw image files were converted to probe set data (*.CEL files) using the Affymetrix GeneChip® Operating Software. Expression microarray data are MIAME compliant. Raw microarray data have been similarly previously generated for the normal *Gprc5a^−/−^* lung cells (Kadara *et al*, in press).

Raw microarray data files (*.CEL) representing the transcriptomes of the *Gprc5a^−/−^* normal and MDA-F471 adenocarcinoma cells were imported and analyzed using the BRB-ArrayTools v.3.7.0 developed by Dr. Richard Simon and BRB-ArrayTools Development Team [25]. Robust multi-array analysis (RMA) was used for normalization of gene expression data using the R language environment [26]. An NNK-ADC (NNK-induced adenocarcinoma) signature was derived which was comprised of gene features that were differentially expressed between the mouse normal *Gprc5a* ^−/−^ cells and MDA-F471 adenocarcinoma cells. The gene features were selected based on the criteria of a p-value<0.001 of a random variance two-sample t-test with permutation and estimation of the false discovery rate and a 2-fold difference in expression. The NNK-ADC signature was also analyzed using Ingenuity Pathways Analysis (IPA®) (http://www.ingenuity.com) to organize the gene features into molecular gene sets and pathways significantly modulated between the *Gprc5a*-knockout normal and NNK-induced adenocarcinoma cells.

The NNK-ADC signature derived from *Gprc5a^−/−^* and MDA-F71 cells constituting lung carcinogenesis in the *Gprc5a* knockout mouse were directly integrated with previously characterized differentially expressed genes (GSE accession #17073) [27] between normal human bronchial epithelial (NHBE) cells and lung tumorigenic 1170-I cells. The tumorigenic cells were derived from a tumor that had developed in nude mice after transplantation of an immortalized cell line derived from NHBE cells and exposed to cigarette smoke condensate *in vivo* [28]. Only orthologous genes present in both mouse and human microarray data and differentially expressed by at least 2-fold between the human NHBE and 1170-I cells (n = 523) (Table S1) were integrated for cluster analysis and functionally analyzed using IPA®. To assess the expression of the NNK-ADC expression signature in human NSCLC compared to normal lung specimens, we integrated unique orthologous members of the mouse gene signature with published human gene expression data from the reports by Su *et al* (27 lung adenocarcinomas and paired adjacent normal lung) [29] and Stearman *et al.* (20 lung adenocarcinomas and 19 adjacent normal lung; from 10 patients in replicate) [30]. The NNK-ADC signature was also integrated with gene expression data of mouse normal lung (n = 15) and adenocarcinoma (n = 29) tissue [30] also available from the study by Stearman *et al.* Raw microarray data from all data sets were analyzed using the BRB-ArrayTools and gene expression data were normalized by RMA. Prior to integration of the NNK-ADC signature with the mouse and human lung cancer data sets, unique orthologous genes and probe sets present in Affymetrix GeneChip® Mouse Genome430 2.0 and the mouse Affymetrix MG-U74Av2 and human Affymetrix HG-U95Av2 and HG-U133A platforms were identified using NetAffx™ from Affymetrix (http://www.affymetrix.com/analysis/index.affx) by searching for orthologous members with their corresponding gene annotations in the platforms. We found 2475 common orthologs of the NNK-ADC gene signature in the U133A dataset from the study by Su *et al.*, and 1877 orthologs and 1452 gene features present in the human HG-U95Av2 and mouse MG-U74Av2 datasets, respectively, obtained from the study by Stearman *et al.* Hierarchical cluster analysis by average linkage was performed with Cluster 2.11, and results were visualized with TreeView programs (Michael Eisen Laboratory, Lawrence Berkeley National Laboratory and University of California, Berkeley; http://rana.lbl.gov/EisenSoftware.htm). Principal component analysis (PCA) in three-dimensional space was also performed using the BRB-ArrayTools software using metric centered correlation.

### Hematoxylin-Eosin (H&E) staining and Immunohistochemistry analysis

Formalin-fixed paraffin-embedded tissue samples were used to prepare 5 µm thick sections. Some sections were stained with Hematoxylin and eosin (H&E) and used for microscopic observation. Other sections were mounted on poly-L-lysine-coated slides and used for immunohistochemical analysis with antibodies against mouse ubiquitin-conjugating enzyme E2C (Ube2c) (Boston Biochem, Cambridge, MA), flap-like endonuclease 1 (Fen1) (Novus Biologicals, Litteleton, CO), minichromosome maintenance 2 (Mcm2) (Cell Signaling Technology, Danvers, MA), and cyclin D1 (Ccnd1) (Cell Marque Rocklin CA). The sections were subjected to antigen retrieval (Target Retrieval Solution, DAKO), and then incubated in peroxidase blocking reagent (DAKO). Sections were then washed with Tris-containing buffer and incubated overnight at 4°C with the primary antibodies. Subsequently, the sections were washed and incubated with the secondary antibody (goat anti-rabbit) using the Evision plus labeled polymer kit (DAKO) for 30 min followed by incubation with avidin-biotin-peroxidase complex (DAKO) and development with diaminobenzidine chromogen for 5 min. Finally, the sections were rinsed in distilled water, counterstained with hematoxylin (DAKO), and mounted on glass slides before evaluation under the microscope. Immunohistochemical analysis of the staining reactivity of the abovementioned markers were done in triplicate on three different histological sections of normal lung or NNK-induced adenocarcinomas. The staining intensities for each antigen were quantified by counting the number of cells exhibiting positive reactivity in up to three microscopic fields per tumor or normal lung in five different *Gprc5a* ^−/−^ mice injected i.p. with NNK. The mean levels of the markers' immunoreactivity were then statistically assessed for significance of differences by the Students *t*-test. P-values less than 0.05 were considered statistically significant.

### Western blotting analysis

Cell monolayers were washed twice with ice-cold PBS, harvested, and processed for western blotting as previously described [31]. The antibodies used for western blotting included Ube2c (Boston Biochem.), Fen1 (B.D. Pharmingen San Diego CA), Mcm2 and cyclin D1 (Cell Signaling Technology), and beta-actin (Sigma Chemical Co.). Antibody binding was detected by the standard enhanced chemiluminescence system. Membranes were stained with beta-actin to ensure equality of protein loading and transfer.

## Results

### NNK exposure increases incidence and multiplicity of lung tumors in *Gprc5a*-knockout mice

**Figure 2:**
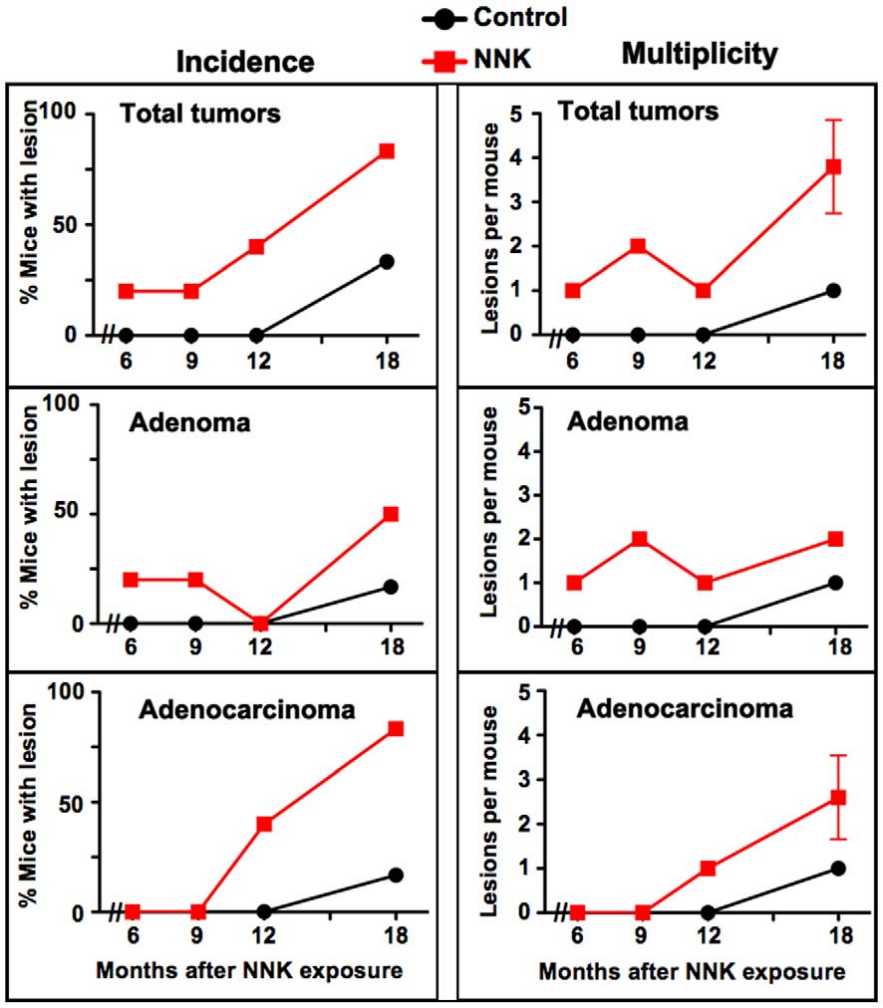
Increased incidence and multiplicity of lung tumors in *Gprc5a*-knockout mice exposed to NNK compared to control mice. Graphs depicting the percentage of mice with lung tumors (left panels) and number of tumors per mouse as a measure of multiplicity (right panels) in the control (orange line) and NNK-exposed (black line) groups at 6, 9, 12 and 18 months following injection with the carcinogen.

The long time required for the development of tumors in the *Gprc5a* ^−/−^ mouse model as well as the low tumor multiplicity had prompted us to determine whether the tobacco carcinogen NNK might enhance lung carcinogenesis in this model. To address this question, we adopted a protocol developed for the A/J mouse model [8], [9], [12], namely 2 months old mice (*n* = 42) received two weekly-spaced i.p. injections of NNK or saline and groups of 5–6 mice each were sacrificed at 6 to 18 months following NNK injection (Fig. 1). Lung lesions were first detected in control mice only 18 months after i.p. injection of saline with one mouse developing an adenoma and another animal an adenocarcinoma. In sharp contrast, lung tumors were detected in 25% of carcinogen exposed mice after as early as 6 months (Fig. 2, left panels). The incidence of tumors increased with time in the NNK-treated *Gprc5a* ^−/−^ mice reaching 50% and 83% for adenomas and adenocarcinomas, respectively at 18 months compared to 16.7% and 16.7%, respectively in the control mice (Fig. 2). Furthermore, NNK increased the multiplicity of both adenomas and adenocarcinomas compared to control mice (Fig. 2, right panels). Assessment of the effects of NNK exposure on total lung lesions' incidence and multiplicity at the longest time period (18 months) revealed 2.5-fold and 4-fold increases, respectively (Fig. 2 upper panels). No lesions were detected in other organs. These findings indicate that exposure of *Gprc5a* ^−/−^ mice to NNK accelerated the development of lung lesions and increased their incidence and number.

### Characterization of lung lesions in NNK-exposed and control *Gprc5a*-knockout mice

**Figure 3:**
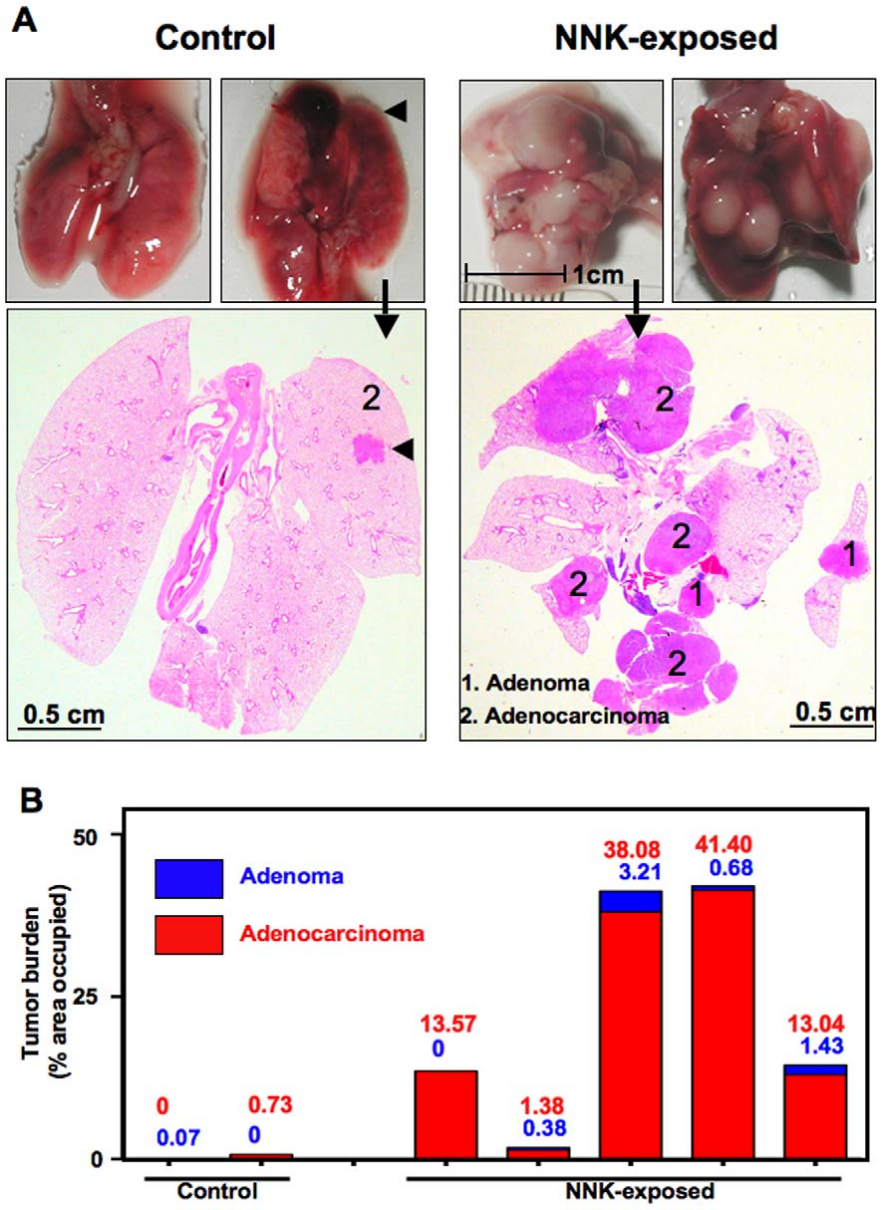
Increased lung tumor burden in NNK-exposed *Gprc5a*-knockout mice. **A**.Gross and macroscopic images representing total lungs excised from two mice each injected i.p with control saline (left) or NNK (right). Arrows indicate gross lung images representing selected H& E stained histological sections of total lungs from both groups and arrowheads indicate presence of a lung tumor (adenomas, 1; adenocarcinomas, 2). **B**. Lung tumor areas were quantified by Image-J software to assess tumor burden displayed in the graph as percentage occupied of total area.

Visual inspection of the lungs clearly revealed that the lesions in mice sacrificed 18 months after NNK were much larger than in controls (Fig. 3A, upper panels). Histological sections of FFPE whole lungs revealed this characteristic also for internal lesions (Fig. 3A, lower panels). We calculated the percent of total lung section area occupied by lesions to estimate tumor burden. The burden of adenocarcinoma lesions in NNK-exposed mice was variable (1.38 to 41.4%) and that of adenoma ranged from 0 to 3.21% (Fig. 3B). In the control mice, where only one adenocarcinoma and one adenoma were found after 18 months, the tumor burdens were 0.73 and 0.07%, respectively.

**Figure 4:**
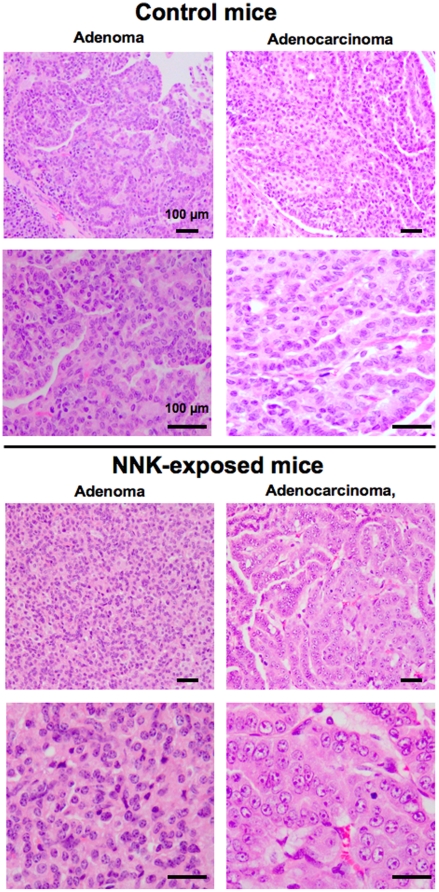
Lung tumors in NNK-exposed *Gprc5a*-knockout mice display increased features of poor differentiation. Representative photomicrographs of tissue histological sections following H&E staining of spontaneous adenomas and adenocarcinomas obtained from control mice (**A**) and from *Gprc5a*-knockout mice exposed to NNK (**B**). Bars, 100 microns.

The diagnosis of individual lesions as adenoma or adenocarcinoma was accomplished after analysis of H&E stained tissue sections of lungs from control and NNK-exposed mice under the microscope and examples of such lesions are presented in Fig. 4. The adenoma and adenocarcinomas lesions from lungs of NNK-exposed mice were composed of cells appearing less differentiated and with higher nuclear crowding and cytological atypia than cells in the corresponding lesions of control mice (Fig. 4). These findings suggested that NNK exposure leads to the development of more aggressive lung tumors compared to control *Gprc5a* ^−/−^ mice.

### Identification of differential gene expression patterns and functional cellular pathways between epithelial cell lines derived from normal lungs of *Gprc5a* ^−/−^ mice and from adenocarcinomas of NNK-exposed mice

**Figure 5:**
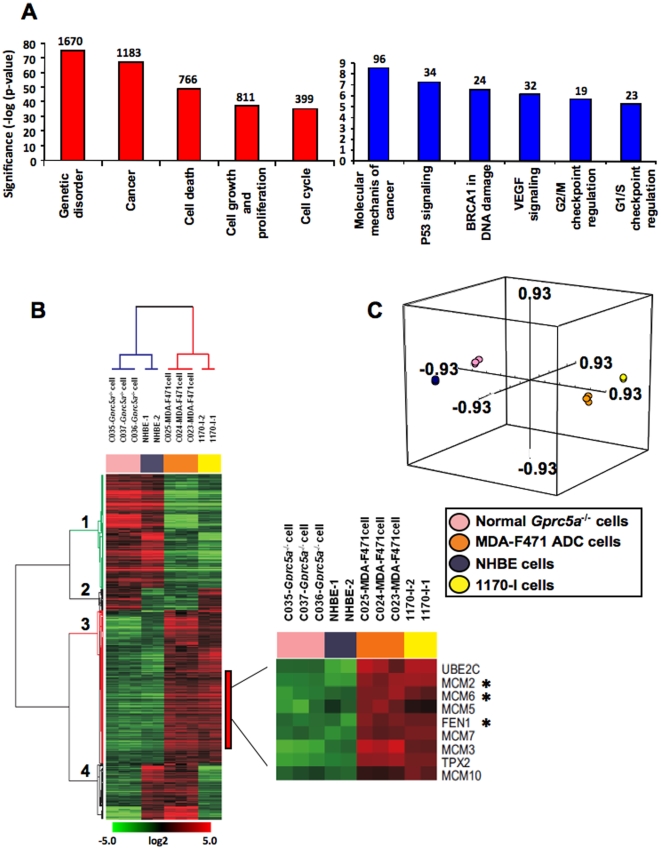
The mouse NNK-ADC signature exhibits significant modulation of cancer-related gene sets and pathways that are similar in an *in vitro* model of human lung carcinogenesis. **A**. The mouse NNK-ADC signature comprising 4981 gene feature differentially expressed between *Gprc5a*-knockout MDA-F471 adenocarcinoma cells and *Gprc5a^−/−^* normal lung cells normal cells was derived based on selection criteria described in the Methods. The signature was functionally analyzed using global functional categories from by IPA®. The value of −log(significance) represents the inverse log of the p-values of the modulation of the depicted functional categories (left panel) and pathways (right panel) between the mouse normal and adenocarcinoma cells. The number of genes displaying more than 2 fold change is indicated above each bar. **B**. Human orthologs of the mouse NNK-ADC and also differentially expressed between the human lung tumorigenic 1170-I and normal NHBE cells (n = 523) were median-centered independently in the mouse and human lung epithelial cells, integrated and then analyzed by hierarchical cluster analysis. High or low gene expression levels are indicated by red or green color, respectively as indicated by the log2 transformed scale bar. Gene clusters are numbered 1 to 4. The clusters of up-regulated and down-regulated genes are highlighted in red and green, respectively. **C**. PCA analysis in three-dimensional space of the integrated data using metric centered correlation.

To begin to understand the mechanism(s) underlying the above effects of NNK, we decided to compare and contrast global gene expression in lung epithelial cells isolated from *Gprc5a^−/−^* mouse trachea and in MDA-F471 tumor cells cultured from an adenocarcinoma from an NNK-exposed *Gprc5a^−/−^* mouse. We derived a gene expression signature signifying differentially expressed gene features between the cell types, NNK-ADC signature. Differentially expressed gene features (n = 4981) were selected based on the criteria of a p<0.001 of the univariate t-test with permutation and estimation of the false-discovery rate and a change in expression of at least 2-fold. Functional analysis of the NNK-ADC signature using IPA® highlighted the significant (significance indicated as −log of the p-values) modulation of several cancer-related molecular function-specific gene sets such as cell death, cell growth and proliferation and cell cycle (all p<0.001) (Fig. 5A, left panel). Moreover, functional analysis revealed canonical cellular pathways that are differentially activated between the adenocarcinoma cells and the normal lung epithelial cells. These pathways included *p53* and vascular endothelial growth factor (*Vegf*) signaling pathways and cell cycle G2/M and G1/S checkpoints (all p<0.001) (Fig. 5A, right panel).

### Differential gene expression patterns between *Gprc5a*-knockout MDA-F471 adenocarcinoma and normal lung cells closely mimic those in an *in vitro* model of human lung carcinogenesis

To assess the relevance of the findings with the murine normal and NNK-induced tumor cells to human lung carcinogenesis, we compared their differential expression patterns to those of a comparable *in vitro* pair of human normal and tobacco-induced tumorigenic lung cells (NHBE and 1170-I, respectively) (refer to Materials and Methods section). Orthologous members of the NNK-ADC signature that were present both in the Affymetrix human genome (HG) U133A and Mouse Genome 430 2.0 platforms and differentially expressed between the NHBE and 1170-I cells (GSE# 17073, [27]) were directly compared and integrated in the mouse and human systems (n = 523). Hierarchical cluster analysis of the integrated data revealed that two gene clusters with similar patterns of differential expression between the mouse and human adenocarcinoma and normal cells (gene cluster 1, red; gene cluster 3, green) (Fig.5B). Although, most genes (81.5%) displayed concordant expression between the mouse and human *in vitro* carcinogenesis systems (Fig. 5B), we observed two clusters of genes with reverse expression in the mouse and human cells; the second and fourth (from the top) gene clusters were comprised of 18 (3.5%) and 84 (16%) genes, respectively, (Fig. 5B). Furthermore, following principal component analysis in three-dimensional space, the mouse MDA-F471 adenocarcinoma cells resided very closely to the human lung 1170-I tumorigenic cells which together were distant from the mouse *Gprc5a^−/−^* normal lung epithelial cells and the human NHBE cells (Fig. 5C). These data demonstrate that gene expression patterns may be conserved between cells in NNK-induced lung carcinogenesis in the *Gprc5a*-knockout mouse and cells constituting an experimental model of human lung carcinogenesis.

**Figure 6:**
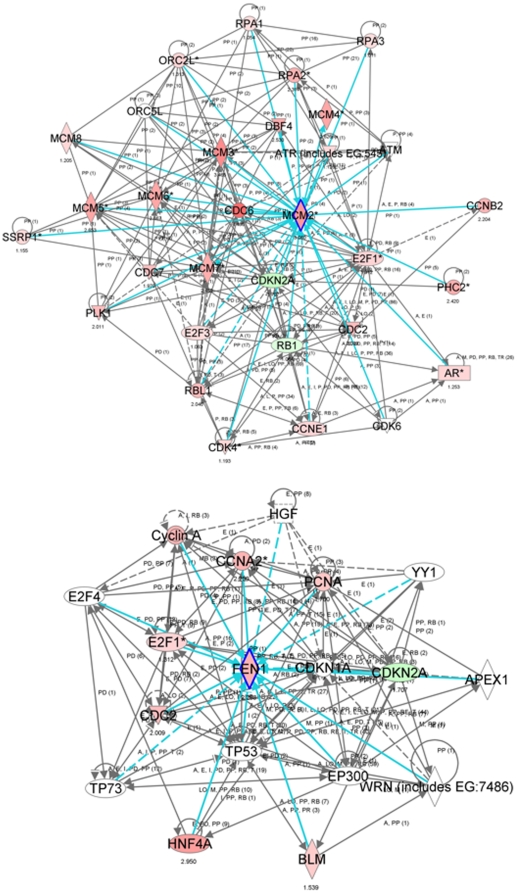
Gene-interaction network analysis of genes differentially expressed between both mouse and human normal lung epithelial and adenocarcinoma cells. IPA® analysis of gene-interaction networks and neighborhoods involving the selected genes, *Mcm2* (top) and *Fen1* (bottom), differentially expressed between *Gprc5a* ^−/−^ normal lung cells and adenocarcinomas as well as between human normal NHBE cells and 1170-I tumorigenic cells (highlighted in inlet of 5**B**). Interaction nodes of the *Mcm2* and *Fen1* genes are highlighted by a blue border. Gene expression variation by at least 2-fold is depicted by color (red, up-regulated; green, down-regulated).

Notably, in both mouse and human carcinogenesis systems, we observed similar differential expression patterns of genes that had been previously characterized by functional pathways analysis in the same human *in vitro* model of lung carcinogenesis [27], namely, *Ube2c*, *Mcm2* and *Fen1* (Fig. 5B, lower right panel). Functional pathways analysis using the IPA® software revealed significant modulation of neighborhood and network gene modules surrounding the *Mcm2* and *Fen1* genes in the *Gprc5a^−/−^* adenocarcinoma (Fig. 6, top and bottom, respectively). The expression levels of *Mcm2* and several related *Mcm* genes such as *Mcms* 3, 4, 5, 6, 7 and 10, as well as *Fen1* were increased (indicated by the red color) in the depicted gene-interaction networks. These findings highlighted genes, e.g. *Fen1* and *Mcm2*, that are predicted to be important in the development of adenocarcinomas in *Gprc5a*-knockout mice exposed to NNK through both their modulation in expression and number and significance of molecular interactions.

### Up-regulation of Ube2c, Mcm2, Fen1 and cyclin D1 proteins in lung adenocarcinoma tissue and cells compared to normal lung in NNK-exposed *Gprc5a*-knockout mice

**Figure 7:**
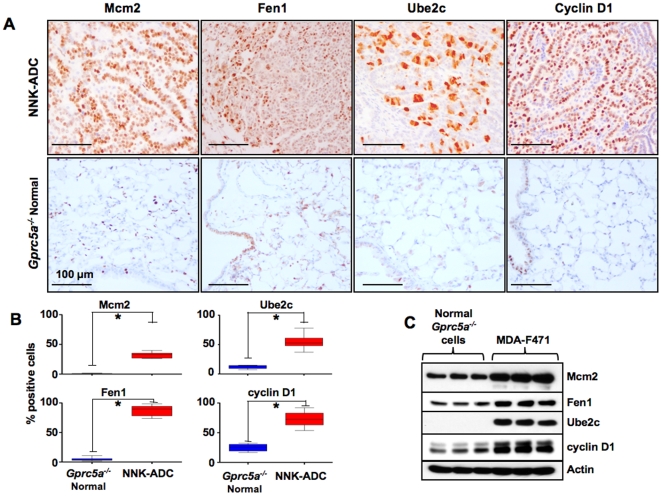
Elevated expression of Ube2c, Mcm2, Fen1 and cyclin D1 proteins in *Gprc5a*-knockout adenocarcinomas and normal lung tissue and cells. **A**. Representative photomicrographs depicting increased expression of the protein products of Mcm2, Ube2c, Fen1 and cyclin D1 in NNK-induced adenocarcinomas and normal lung histological tissue specimens obtained from NNK-exposed *Gprc5a*-knockout mice. All four proteins exhibited mainly nuclear localization of expression. **B**. The number of cells exhibiting positive reactivity (nuclear staining) was counted in up to three separate microscopic fields per tumor or normal lung in five different NNK-exposed *Gprc5a* ^−/−^ mice. P-values were obtained by the students two-sample *t*-test (*, p<0.001). **C**. Western blotting analysis of the four antigens in three different culture dishes of each of the *Gprc5a* ^−/−^ MDA-F471 adenocarcinoma and normal lung cells. Approximately 10^6^ cells per 10 cm cell culture dish were harvested to prepare total protein extracts. Samples of these extracts (20 µg protein) were analyzed by western blotting using primary antibodies against the indicated proteins. Membrane blots were also analyzed with antibodies against β-actin to compare protein loading in the different lanes.

Because gene expression at the mRNA level does not always correspond to protein levels, we then examined the expression of the protein products of *Ube2c*, *Fen1* and *Mcm2* in *Gprc5a* ^−/−^ lung normal and adenocarcinoma tissue and cells. We also assessed the protein levels of the cell cycle marker, *cyclin D1* in the same tissue and cells because it was up-regulated in the MDA-F471 cells at the mRNA level and was previously shown to display intensive expression in lung adenomas and adenocarcinomas from mice exposed to NNK [10]. Immunohistochemical analysis demonstrated the up-regulation of the protein levels of *Mcm2*, *Fen1*, *Ube2C* and *cyclin D1* in lung adenocarcinoma tissue of NNK-exposed *Gprc5a*-knockout mice compared to the levels in normal lung tissue obtained from the same mice (Fig. 7A). The mean levels of the immunoreactivity of were all statistically significantly higher in lung adenocarcinoma relative to normal lung tissue in the NNK-exposed mice (p<0.001, *) (Fig. 7B). In accordance, Mcm2, Fen1, Ube2c and cyclin D1 proteins were also relatively higher in the MDA-F471 mouse adenocarcinoma cells compared to the normal *Gprc5a^−/−^* cells as revealed by the western blotting analysis (Fig. 7C). These results demonstrate that there is a concordance between the mRNA and protein up-regulation of the above markers when comparing both tumor and normal cells and tissues.

### Integrated cluster analysis using the NNK-ADC expression signature effectively separates mouse and human NSCLC tissue from normal lung

**Figure 8:**
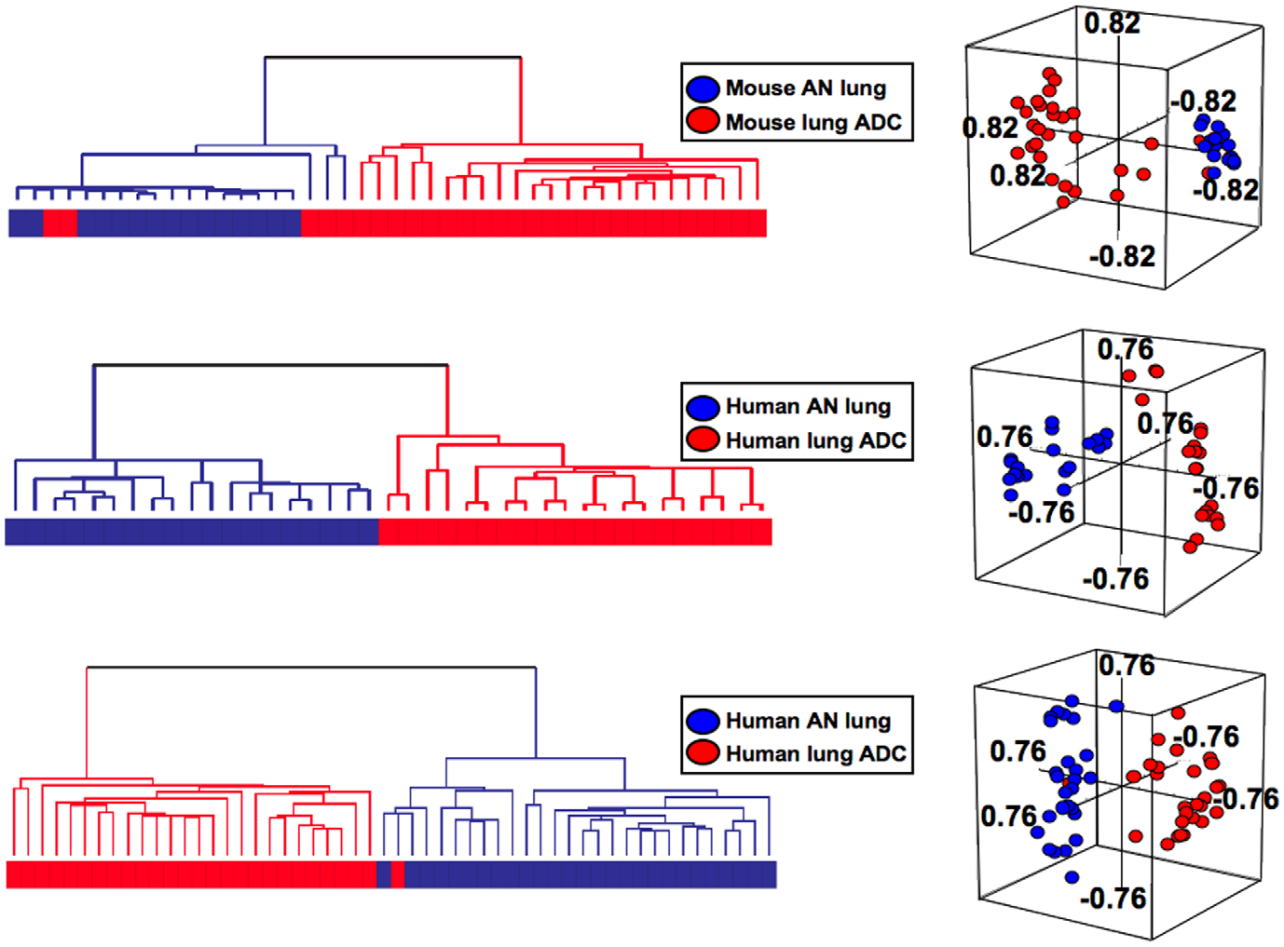
The mouse NNK-ADC expression signature effectively separates both mouse and human lung adenocarcinomas from their adjacent normal counterparts. **A**. Dendogram (left) of hierarchical cluster analysis and PCA (right) of the gene features (n = 1452) of the NNK-ADC signature that are present in the Affymetrix MG-U74Av2 platform and gene expression data of mouse normal lung (n = 15) and adenocarcinoma (n = 29) tissue from the study by Stearman *et al* [30]. Dendograms (left) of hierarchical cluster analysis and PCA (right) of human datasets including 20 lung adenocarcinomas and 19 adjacent normal lung samples from the study by Stearman *et al* (**B**) and 27 lung adenocarcinomas and 27 paired adjacent normal lung from the study by Su *et al* [29] (**C**) and using only orthologous genes of the signature and found in the Affymetrix MG-U95Av2 (**B**, n = 1877) and HG-U133A (**C**, n = 2475) platforms.

We sought to test the relevance of the differential gene expression patterns between the *Gprc5a*-knockout MDA-F471 adenocarcinoma and normal lung epithelial cells to the gene expression patterns in publicly available microarray datasets of both mouse and human NSCLC and normal lung tissue. We analyzed the NNK-ADC signature in the expression data of mouse lung adenocarcinomas (n = 29) and adjacent normal lung samples (n = 15) from the study by Stearman *et al* [30]. Hierarchical clustering analysis of the data revealed that the NNK-ADC signature effectively separated mouse normal lung tissues from tumors indicating that the NNK-ADC signature was highly associated with mouse adenocarcinomas (Fig. 8A). By using only orthologous genes present in both mouse and human microarray platforms, the NNK-ADC signature was also integrated with gene expression data of human lung adenocarcinomas and adjacent normal counterparts available from previous studies [29], [30]. Hierarchical clustering analysis of the data revealed that the signature was highly expressed in human tumors as it effectively human lung adenocarcinomas from normal lung tissues (Figs. 8B and 8C). Moreover, PCA analysis of all integrated analyses revealed that lung adenocarcinomas lay considerably distant from normal lung in three-dimensional space (Figs. 8A, 8B and 8C). In addition, the differences in the number of lung adenocarcinomas and normal lung tissues between the two identified clusters were all statistically significant (p<0.001 by the Fisher's test). These findings demonstrate that differential gene expression patterns between *Gprc5a*-knockout NNK-exposed MDA-F471 adenocarcinoma and normal lung cells may resemble lung tumor molecular profiles in other mouse models of lung carcinogenesis (e.g., urethane-exposed A/J mice, [30]) and in human lung adenocarcinomas.

## Discussion

Previously, we have identified the *Gprc5a* gene as a mouse lung-specific tumor suppressor after finding that mice lacking both alleles of this gene develop spontaneous lung adenomas and adenocarcinomas unlike their wild-type littermates. Tumor development in this model was preceded by a long latency period of up to 24 months [20]. Given that most human lung cancers (80%) occur in ever smokers [2], [4], [5], we thought that it might be valuable to investigate the effects of exposure of *Gprc5a*-knockout mice to a tobacco-specific carcinogen such as NNK. In this study we found that exposure of young mice to NNK (only two i.p. injections separated by one week) shortened the time to tumor development and increased the incidence, multiplicity, and size of both adenomas and adenocarcinomas in *Gprc5a*-knockout mice compared to control mice. In this design, the exposure of mice to the lung specific carcinogen occurs early in life suggesting that the model mimics events that can be expected to occur in former smokers. We based the design of the study with the *Gprc5a*-knockout mice on previous studies with strain A/J mice [8], [9], [12], which also have a predisposition for spontaneous lung tumors [11] and exhibit enhanced carcinogenesis after i.p. injection of NNK [11]. Our model is distinct from the A/J model in that our mice have a defined genetic lesion, namely loss of *Gprc5a* [20], whereas the genetic lesion in A/J mice is not fully defined. Interestingly, a pulmonary adenoma susceptibility 1 (*Pas1*) locus has been found in A/J mice on chromosome 6. This locus was found to include two genes, *Kras*2 (Kirsten rat sarcoma oncogene 2) and *Casc1* (cancer susceptibility candidate 1), that are strong candidates for mediating the predisposition to adenoma in the A/J mice [32]. It is noteworthy that the *Gprc5a* gene is also located on mouse chromosome 6 (G1), however, it is located 10 Kb away from the *Kras2* gene and is not encompassed within the *Pas1* locus.

The exposure to NNK shortened the time to tumor development and markedly increased the incidence and burden of adenocarcinomas. In our study, the tumor burden among the five mice sacrificed at the 18 month time point was variable, however, even the smallest adenocarcinoma among the NNK-exposed mice was larger than the single spontaneous adenocarcinoma. The incidence of adenocarcinomas in the NNK exposed *Gprc5a* knockout mice (80%) was much higher than the 17% incidence of spontaneous adenocarcinomas in mice followed for up to 24 months [20] and also higher than that reported previously by others for NNK-treated A/J mice [33]. A histological examination of features of the spontaneous adenoma and adenocarcinoma and their comparison to the corresponding NNK-induced tumors indicated that the tumors induced by the tobacco carcinogen appear to be less differentiated and more aggressive. However, it is not possible to draw statistically significant conclusions about the differential tumor histology because we only observed one spontaneous adenoma and one spontaneous adenocarcinoma in the control mice compared to numerous tumors in the NNK group. Nonetheless, our model is relevant to human lung cancer because the incidence of adenocarcinoma among human patients is increasing relative to squamous cell carcinomas [4]. The development of adenocarcinomas at the exclusion of squamous cell carcinomas is characteristic of other mouse lung carcinogenesis models including NNK-treated A/J strain mice [9], [12], [23] and mutant *Kras* transgenic mice [16]. In future studies, it would be worthwhile to investigate the tumor incidence of *Gprc5a* knockout mice exposed to varying and increases doses of NNK.

To begin to understand the molecular mechanisms underlying the development of adenocarcinomas in NNK-exposed *Gprc5a*-knockout mice, we used microarray profiling, to compare and contrast the transcriptomes of MDA-F471 malignant cell line and normal lung epithelial cells isolated from NNK-exposed *Gprc5a* ^−/−^ mice and from normal tracheas of untreated *Gprc5a* ^−/−^ mice, respectively. This comparison led us to derive a gene expression signature, which we named NNK-ADC signature that is comprised of genes differentially expressed between the mouse adenocarcinoma and normal cells. Upon functional pathways analysis, these genes were organized into significantly differentially activated prominent cancer-related pathways such as G1/S and G2/M cell cycle checkpoints and cell growth and proliferation. The perturbation of such cellular pathways may explain the enhanced carcinogenesis in NNK-exposed *Gprc5a* ^−/−^ mice as cell cycle promoting genes have been implicated in NNK-mediated tumorigenesis in A/J strain mice [10].

Comparative genomic approaches have shown promise to highlight mouse models reliably mirroring the corresponding human disease [34], [35] and to unravel evolutionarily conserved and important genes [36]. A pertinent question is whether the differential gene expression patterns between the *Gprc5a* ^−/−^ adenocarcinoma MDA-F471 cells and the normal lung *Gprc5a* ^−/−^cells are relevant to human lung carcinogenesis. To explore this question, we first integrated orthologous features of the NNK-ADC signature derived herein with previously characterized genes, which we had found recently to be differentially expressed [27] between human normal (NHBE) and tumorigenic (1170-I) cells. Our integrated cluster analysis revealed a substantial overlap between both pairs of normal and malignant cells with most genes differentially expressed in a similar pattern in both mouse and human mRNA profiles. Further use of comparative genomic approaches and integrating the NNK-ADC signature in published microarray data sets of both mouse and human normal lung and adenocarcinoma tissue, the signature was found to effectively discriminate lung tumor tissues from their adjacent normal counterparts. These data demonstrate that the differential gene expression patterns between *Gprc5a*-knockout NNK-induced lung adenocarcinoma and normal epithelial cells may be conserved between mouse and human lung epithelial cells.

Using comparative functional genomics and functional pathways analysis followed by validation at the protein level by immunohistochemistry and western blotting analyses, we highlighted the marked up-regulation of *Ube2c*, *Mcm2* and *Fen1* in *Gprc5a*-knockout mouse adenocarcinoma relative to normal lung. Interestingly, the expression of these genes has been linked to human lung cancer. *UBE2C*, a ubiquitin conjugating enzyme, is important for lung cancer cell survival [37] and was previously shown by our group to increase in expression progressively in lung lesions [27]. *MCM2* is a component of the minichromosome maintenance complex that was demonstrated to be highly expressed in human NSCLC as well as in preneoplastic lesions [38], [39]. The flap structure-specific endonuclease, *FEN1*, was reported to exhibit elevated expression in human NSCLC cells compared to normal human bronchial epithelial cells [40]. It is noteworthy that we have previously demonstrated the progressive and significant modulation of the mRNA levels of *UBE2C*, *MCM2* and *FEN*1 in normal, immortalized, transformed and tumorigenic lung epithelial cells constituting an *in vitro* model of human lung carcinogenesis [27]. Moreover, the transformed and tumorigenic derivatives of this human *in vitro* model were generated following exposure of xenotransplants of BEAS-2B cells (derived from NHBE cells immortalized with the large T antigen of SV40) to another tobacco-related carcinogen, cigarette smoke condensate [28]. Therefore, it is plausible to suggest that the association of expression of *UBE2C*, *MCM2* and *FEN1* with tobacco-induced lung carcinogenesis appears to be evolutionarily conserved. Indeed, we have previously unraveled a robust association between UBE2C protein expression and smoking habits or patterns in histological tissue specimens obtained from approximately 300 NSCLC patients [27]. These findings evoke the possibility that the characterized NNK-ADC signature signifying molecular differences between the *Gprc5a*-knockout NNK-induced adenocarcinoma and normal lung cells may be valuable in the discovery of genes that are relevant to tobacco-induced human lung cancer cells.

Although we had been able to isolate only a single pair of *Gprc5a* ^−/−^ normal lung epithelial cells and lung adenocarcinoma cells from an NNK-exposed mouse, we were successful in deriving an NNK-ADC expression signature. We recognize that using an expression signature signifying lung adenocarcinoma development in the NNK-exposed *Gprc5a* knockout mouse model based on a single pair or normal and tumor cells and without a complimentary set of data from another pair of samples represents a potential limitation. However, it is important to mention that when we integrated the genes differentially expressed between the mouse *Gprc5a* ^−/−^ normal and MDA-F471 tumor cells with those modulated between human normal and tumorigenic lung cells, most (81.5%) exhibited concordant expression between the *in vitro* carcinogenesis systems from both species. Moreover, the protein expression of several key genes of the NNK-ADC signature was validated at the protein level by immunohistochemistry in histological tissue specimens of normal lung and adenocarcinomas obtained from five *Gprc5a* knockout mice. Furthermore, the NNK-ADC expression signature was analyzed in publicly available datasets and was found to effectively separate human lung adenocarcinomas from normal lung tissues. Therefore, it is likely that gene expression patterns in the NNK-exposed *Gprc5a* knockout mouse model closely resemble those found in human lung cancer epithelial cells.

In conclusion, we have found that treatment of *Gprc5a*-knockout mice with the tobacco-specific carcinogen NNK increased the rate of lung adenocarcinoma development compared to the spontaneous rate in control mice. Moreover, we were able to highlight genes potentially important for NNK-induced lung carcinogenesis in the *Gprc5a*-knockout mouse. We also provide evidence that suggests that differential gene expression patterns in NNK-induced lung carcinogenesis in the *Gprc5a*-knockout mouse cells may be conserved in human lung cancer epithelial cells. Further experimentation is warranted to validate the reliability of the NNK-exposed *Gprc5a*-knockout mouse for the study of tobacco carcinogen(s)-induced lung tumorigenesis and for use in future chemoprevention studies.

## Supporting Information

Table S1 (0.15 MB XLS)
